# Feasibility of a Multimodal Prehabilitation Programme in Patients Undergoing Cytoreductive Surgery for Advanced Ovarian Cancer: A Pilot Study

**DOI:** 10.3390/cancers14071635

**Published:** 2022-03-23

**Authors:** Berta Diaz-Feijoo, Nuria Agusti-Garcia, Raquel Sebio, Antonio López-Hernández, Marina Sisó, Ariel Glickman, Nuria Carreras-Dieguez, Pere Fuste, Tiermes Marina, Judit Martínez-Egea, Laura Aguilera, Juan Perdomo, Amaia Pelaez, Manuel López-Baamonde, Ricard Navarro-Ripoll, Elena Gimeno, Betina Campero, Aureli Torné, Graciela Martinez-Palli, María J. Arguis

**Affiliations:** 1 Gynecologic Oncology Unit, Clinic Institute of Gynecology, Obstetrics, and Neonatology, Hospital Clinic of Barcelona, Institut d’Investigacions Biomèdiques August Pi i Sunyer (IDIBAPS), Universitat de Barcelona, 08036 Barcelona, Spain; nagusti@clinic.cat (N.A.-G.); glickman@clinic.cat (A.G.); ncarreras@clinic.cat (N.C.-D.); pfuste@clinic.cat (P.F.); marina@clinic.cat (T.M.); jumartinez@clinic.cat (J.M.-E.); laguilera@clinic.cat (L.A.); atorne@clinic.cat (A.T.); 2 Department of Rehabilitation, Hospital Clinic de Barcelona, 08036 Barcelona, Spain; sebio@clinic.cat (R.S.); elgimeno@clinic.cat (E.G.); 3 Anesthesiology and Intensive Care, Hospital Clinic de Barcelona, Institut d’Investigacions Biomèdiques August Pi i Sunyer (IDIBAPS), Universitat de Barcelona, 08036 Barcelona, Spain; lopez15@clinic.cat (A.L.-H.); jmperdomo@clinic.cat (J.P.); ampelaez@clinic.cat (A.P.); lopez10@clinic.cat (M.L.-B.); rnavarr1@clinic.cat (R.N.-R.); gmartin@clinic.cat (G.M.-P.); mjarguis@clinic.cat (M.J.A.); 4 Nutrition and Clinical Dietetics, Hospital Clinic de Barcelona, 08036 Barcelona, Spain; siso@clinic.cat (M.S.); bcampero@clinic.cat (B.C.); 5 Biomedical Research Networking Center on Respiratory Diseases (CIBERES), 28029 Madrid, Spain

**Keywords:** multimodal prehabilitation, Enhanced Recovery After Surgery, advanced ovarian cancer, surgical complications, length of stay, comprehensive complications index

## Abstract

**Simple Summary:**

Multimodal prehabilitation programmes represent an innovative approach to promoting surgical recovery by improving the physiological and psychological baseline resilience in conjunction with nutritional optimisation in order to reduce the stress to which the patient is subjected during surgery. These programmes are becoming widespread in different fields of surgery, but in major gynaecological surgery for ovarian cancer, there is still no clear consensus. In this study, we aimed to assess the feasibility of these interventions and their impact on postoperative outcomes in women with advanced ovarian cancer. All patients received perioperative care in accordance with Enhanced Recovery After Surgery guidelines. This pilot study showed that multimodal prehabilitation before surgery is feasible and safe, since we observed good adherence without any major adverse effects in this vulnerable population. Additionally, we found that prehabilitation reduced hospital length of stay and the time from surgery to adjuvant chemotherapy.

**Abstract:**

Introduction: Treatment for advanced ovarian cancer (AOC) comprises cytoreductive surgery combined with chemotherapy. Multimodal prehabilitation programmes before surgery have demonstrated efficacy in postoperative outcomes in non-gynaecological surgeries. However, the viability and effects of these programmes on patients with AOC are unknown. We aimed to evaluate the feasibility and postoperative impact of a multimodal prehabilitation programme in AOC patients undergoing surgery. Methods: This single-centre, before-and-after intervention pilot study included 34 patients in two cohorts: the prehabilitation cohort prospectively included 15 patients receiving supervised exercise, nutritional optimisation, and psychological preparation from December 2019 to January 2021; the control cohort included 19 consecutive patients between January 2018 and November 2019. Enhanced Recovery After Surgery guidelines were followed. Results: The overall adherence to the multimodal prehabilitation programme was 80%, with 86.7% adherence to exercise training, 100% adherence to nutritional optimisation, and 80% adherence to psychological preparation. The median hospital stay was shorter in the prehabilitation cohort (5 (IQR, 4–6) vs. 7 days (IQR, 5–9) in the control cohort, *p* = 0.04). Differences in postoperative complications using the comprehensive complication index (CCI) were not significant (CCI score: 9.3 (SD 12.12) in the prehabilitation cohort vs. 16.61 (SD 16.89) in the control cohort, *p* = 0.08). The median time to starting chemotherapy was shorter in the prehabilitation cohort (25 (IQR, 23–25) vs. 35 days (IQR, 28–45) in the control cohort, *p* = 0.03). Conclusions: A multimodal prehabilitation programme before cytoreductive surgery is feasible in AOC patients with no major adverse effects, and results in significantly shorter hospital stays and time to starting chemotherapy.

## 1. Introduction

The standard treatment for advanced ovarian cancer (AOC) consists of complete cytoreductive surgery combined with chemotherapy. This surgery implies performing complex procedures with a high risk of serious complications often resulting in functional decline and directly determining postsurgical recovery and survival [1]. Grade III or IV postoperative complications [2] occur in over 20% of patients undergoing surgery for AOC, and up to 5% of these complications result in the death of the patient [1,3,4,5,6]. As a consequence, postoperative complications can lead to delays in initiating chemotherapy, which are directly related to decreased survival [7]. 

Growing evidence indicates that postoperative organ dysfunction secondary to surgical stress response is a fundamental cause of postoperative morbidity and mortality [8,9]. The Enhanced Recovery After Surgery (ERAS^®^) programme aims to reduce perioperative morbidity and decrease the length of hospital stays without increasing readmission rates [10]. However, ERAS^®^ protocols mainly focus on perioperative and postoperative care, disregarding the assessment of presurgical risk factors. 

Recent studies have shown that multimodal prehabilitation programmes, including extensive preoperative evaluation, nutritional and psychological strategies, and physical intervention, have a positive impact on postoperative functional outcomes in other non-gynaecological major abdominal surgeries [11,12,13,14]. To our knowledge, strong evidence of the beneficial effects of prehabilitation in gynaecological interventions is lacking [15]; the only available published information on prehabilitation programmes in patients with gynaecologic cancer comes from a single case report [16].

Thus, we aimed to evaluate the feasibility and effectiveness of a multimodal prehabilitation programme in patients undergoing surgery for AOC. 

## 2. Materials and Methods

### 2.1. Study Design

This was a single-centre, before-and-after intervention pilot study, including patients who underwent surgery for AOC (FIGO stage IIIC or IV) at the Hospital Clinic of Barcelona. Patients were stratified into two cohorts based on the implementation of multimodal prehabilitation programmes: the prehabilitation cohort included consecutive patients between December 2019 and January 2021, and the historical cohort of controls included consecutive patients who underwent surgery at the same hospital between January 2018 and November 2019, without performing multimodal prehabilitation programmes. 

Eligible patients fulfilled the following characteristics: patients with AOC who underwent primary cytoreductive surgery (PCS) or interval debulking surgery (IDS) after 3 or 4 cycles of chemotherapy, or patients with recurrent ovarian cancer who underwent secondary or tertiary surgery. Patients in the prehabilitation cohort were preoperatively scheduled allowing for at least 2 weeks for the prehabilitation intervention. Patients were excluded if they were classified as Eastern Cooperative Oncology Group (ECOG) performance status ≥ 2 and had premorbid conditions (i.e., cardiorespiratory, musculoskeletal, and/or neurological limitations) that contraindicated exercises and fitness assessments. We also excluded patients in the prehabilitation cohort who refused to participate in the prehabilitation programme.

Institutional board approval for the project was previously obtained (HCB/2020/0317) and all patients in the prehabilitation cohort provided written informed consent. The data collected were entered by research staff members into the Research Electronic Data Capture (REDCap) programme via a secure webpage interface.

### 2.2. Enhanced Recovery after Surgery Program

This protocol was applied to both the prehabilitation and control groups. Key aspects of this protocol include: prevention of prolonged fasting allowing oral intake of clear fluids up to 2 h before induction of anaesthesia; preoperative administration of oral carbohydrates 2–3 h before surgery; preoperative use of oral antibiotics and avoidance of mechanical bowel preparation except if a bowel resection was planned; administration of thromboprophylaxis; pre-, intra-, and postoperative euvolemia via goal-directed fluid therapy; maintenance of normothermia; intraoperative and postoperative opioid-sparing multimodal analgesia; avoidance or early removal of surgical drains; early removal of the urine catheter; and early ambulation and feeding. 

### 2.3. Prehabilitation Cohort: Multimodal Prehabilitation Programme

In addition to standard care based on ERAS^®^ protocols [10], patients in the prehabilitation cohort underwent a multimodal prehabilitation programme aimed at improving physical activity, nutrition, and psychological readiness. Patients performed 2 to 4 weeks of prehabilitation. The duration of the prehabilitation programme was adapted to the time available in the surgical schedule.

**Figure 1:**
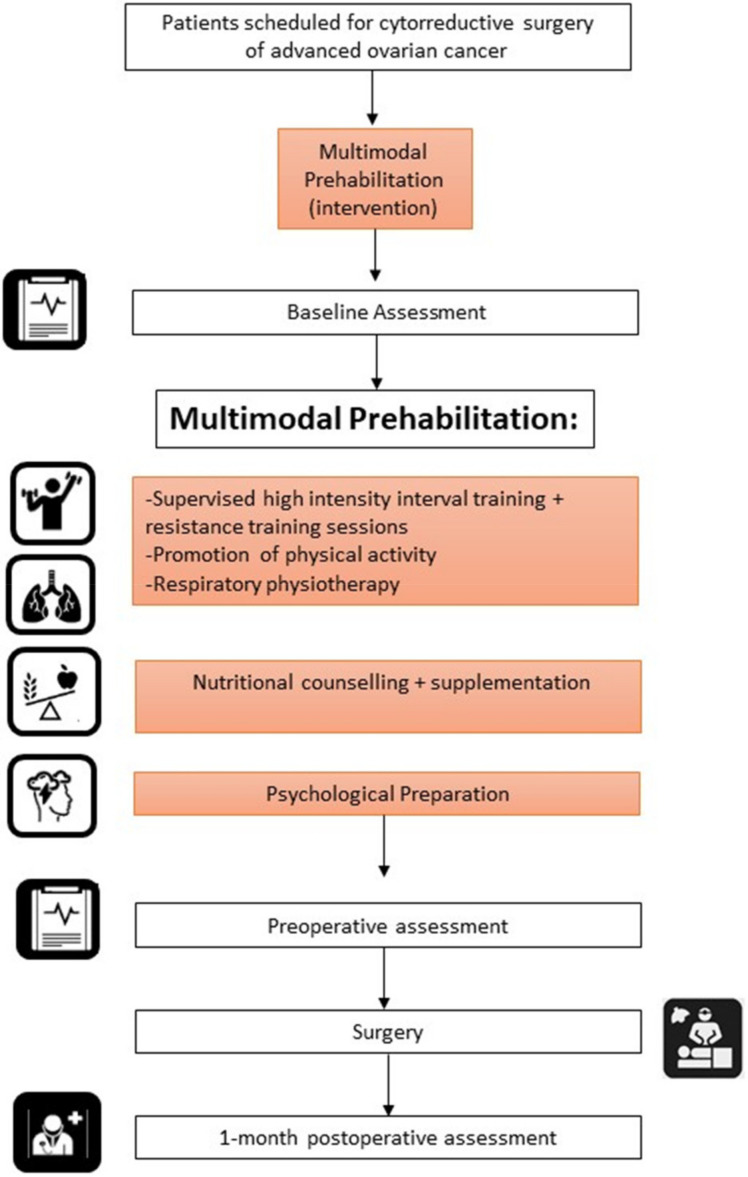
Flow diagram showing the timing of patient assessments and interventions in the prehabilitation cohort.

Quality-of-life measurements were evaluated with the QLQ-C30 questionnaire. Physical activity was measured by the 6-minute walking test and the Yale Physical Activity Survey. Nutritional status was assessed by a clinical dietitian using the Global Leadership Initiative on Malnutrition criteria, and disability and mental state were evaluated using the Hospital Anxiety and Depression Scale (HADS) (Figure 1). We performed baseline, preoperative, and 1-month postoperative assessments. The postoperative evaluation was performed just one month after surgery because chemotherapy is usually scheduled four weeks after the intervention and a later evaluation might limit the results of the tests. 

To improve physical activity, patients underwent a supervised individual exercise programme comprising 3 weekly sessions of endurance and resistance training in our hospital gym. Each session comprised high-intensity aerobic training on a cycloergometer or treadmill and strength training. Endurance training consisted of a 5 min warm-up followed by 30 min of high-intensity interval training and a 5 min cool-down; the workload was set according to the patient’s tolerance using the Borg Rating of Perceived Exertion scale [17]. Strength training consisted of 3 sets of 10 to 15 repetitions of three exercises: horizontal row, chest press, and quadriceps bench. Moreover, patients received a fitness tracker. Physical activity goals were established during a motivational interview on the first day of the prehabilitation programme and adjusted weekly according to the patient’s attainment. To prevent possible postoperative respiratory complications, patients underwent respiratory physiotherapy with a volume-oriented incentive spirometer. A physiotherapist was in charge of monitoring patient complications during physical exercise.

To improve nutritional status, a registered dietitian elaborated a nutritional intervention plan after assessing the patients’ initial status based on the global leadership initiative on malnutrition criteria [18], bioimpedance analysis (InBody 770 Body Composition Analysers), and a blood test including parameters that reflect nutritional status. Prealbumin was evaluated after performing the prehabilitation programme in the prehabilitation cohort. The plan aimed to ensure that the patients’ caloric and protein requirements were met through diet and supplementation with whey protein isolate (Fresenius^©^) when necessary, targeting ingestion of 1.6–2 g protein/kg body weight/day to stimulate muscle mass synthesis. One week before surgery, patients were administered an immunomodulatory formula with specific nutrients such as arginine, omega-3 fatty acids, glutamine, and antioxidants.

A clinical psychologist used the HADS [19] to assess the psychological state of the patients, referring patients with comorbid psychopathology to a specialised service, and personal interviews to assess coping strategies and motivation to engage in the physical activity and nutritional programmes. To boost patient motivation and learn coping strategies to manage symptoms of anxiety and depression, patients attended cognitive behavioural group sessions. During the COVID-19 outbreak, these sessions were replaced by individual online sessions. These sessions were complemented with psychoeducational material and audio guides which promoted physical activity through a health mobile application (preHAB^®^).

Adherence to the multimodal prehabilitation programme was deemed satisfactory when the patient completed ≥ 6 exercise training sessions or ≥75% of the scheduled exercise training sessions, fulfilled ≥ 75% of protein supplementation intake and immunomodulatory formula before surgery, and attended ≥1 session with the psychologist.

### 2.4. Control Cohort

This cohort followed enhanced recovery protocols [10] and preoperative measures established in our hospital, such as general nutritional advice, physical activity recommendations, and advice to stop smoking and alcohol intake. 

### 2.5. Basal Health Status Assessment

To assess the frailty status of our population, we used the Charlson Comorbidity Index and the ECOG scale in both the prehabilitation and control groups. 

### 2.6. Surgery and Postoperative Follow-Up Assessment

All cytoreductive surgeries were performed by experienced gynaecologic oncologists, who used the Aletti score to estimate surgical complexity [20] and the European Society of Gynaecological Oncology’s Ovarian Cancer Operative Report to calculate the peritoneal cancer index (PCI) [21]. The surgical procedures in the historical cohort and the prehabilitation cohort did not differ. Postoperative complications were recorded, assessed with the Clavien–Dindo classification [2], and summarised with the comprehensive complication index (CCI) [22]. Intensive care unit (ICU) stay, length of hospital stay, and complications within 30 days after surgery were also recorded. Discharge criteria were defined as the ability to tolerate oral feeding, good pain control with oral analgesia, and tolerance for ambulation. The time from surgery to adjuvant chemotherapy was defined as the period from surgery to the first chemotherapy administration.

### 2.7. Statistical Analysis

Results are presented as mean (standard deviation (SD)) or median (interquartile range (IQR)) for quantitative variables, based on the distribution of the data. Categorical variables are reported using relative and absolute frequencies (percentages). To compare continuous variables, we used Student’s *t*-test or the Mann–Whitney U-test, as appropriate. To compare categorical variables, we used the chi-square test or Fisher’s exact test, as appropriate. We also used univariate regression analysis to compare characteristics and oncological outcomes of patients with AOC treated with or without multimodal prehabilitation. All tests were 2-tailed with an alpha-risk of 0.05. Imputation of missing values was not performed. The STATA statistical programme (STATA v.15.0; StataCorp LLC, College Station, TX, USA) was used for data analysis.

## 3. Results

Forty patients were assessed for eligibility: 20 patients in the prehabilitation cohort and 20 patients in the control cohort. Of the 20 patients initially assessed for eligibility to receive prehabilitation, four declined participation and another was excluded because there was not sufficient time to perform the programme from the day of diagnosis until the day of the scheduled surgery (<2 weeks). Of the 20 patients initially assessed for eligibility in the control cohort, one patient was ruled out after performing a diagnostic laparoscopy to assess resectability for an IDS. There were no patients lost to follow-up 1 month after the intervention. Thus, 15 patients were analysed in the prehabilitation group and compared with the 19 patients of the control group.

### 3.1. General Characteristics

The proportion of patients who required PCS or IDS was not significantly different between groups (*p* = 0.21) (Table 1). The two cohorts were similar according to baseline, clinical, and surgical characteristics such as age, PCI, and Aletti score.

### 3.2. Adherence to The Prehabilitation Program

Adherence to the multimodal prehabilitation programme was satisfactory in 12 (80%) patients. Adherence to physical training was satisfactory in 13 (86.7%) patients; logistic issues due to the COVID-19 outbreak prevented two patients from achieving satisfactory adherence. The median duration of the prehabilitation programme was 2 weeks (2,3), and patients attended a median of 6 (4–8) supervised exercise training sessions. The median adherence per person to the training sessions was 83% (60–100). Three patients reported mild dizziness during sessions that did not prevent them from continuing the session. Regarding the nutritional programme, adherence to protein supplementation and the immunomodulatory formula was ≥75% in all patients. (Table 2). Adherence to psychological sessions was satisfactory in 12 (80%) patients. 

### 3.3. Surgical Results

The median length of hospital stay was shorter in the prehabilitation cohort (5 days (4–6)) vs. 7 days (5–9), *p* = 0.04). The median ICU stay was shorter in the prehabilitation cohort, but did not reach statistical significance between the two cohorts (*p* = 0.88). After a nutritional intervention plan in the prehabilitation cohort, we observed that preoperative prealbumin levels were higher (0.235 (0.214–0.316) g/L vs. 0.1805 (0.130–0.230) g/L in the control cohort, *p* < 0.01). One patient (6.7%) required blood transfusion in the prehabilitation cohort. Six intraoperative complications (31.6%) occurred in the control cohort: one patient had an intraoperative intestinal injury and five other patients required blood transfusion. The mean CCI scores were not significantly different (9.3 (12.12) in the prehabilitation cohort vs. 16.61 (16.89) in the control cohort, *p* = 0.08). Postoperative complications occurred in six patients (40%) in the prehabilitation cohort and in 12 patients (63.15%) in the control cohort (*p* = 0.30); Clavien–Dindo grade III complications were observed in five patients (26.3%) in the control cohort, but in none of those in the prehabilitation cohort. The median time to starting chemotherapy was shorter in the prehabilitation cohort (25 days (23–25) vs. 35 days (28–45), *p* = 0.03). Table 3 shows all the surgical variables and outcomes. 

## 4. Discussion

### 4.1. Summary of Main Results

In this pilot study, a multimodal prehabilitation programme consisting of high-intensity physical exercise, together with nutritional supplementation and psychological support measures before cytoreductive surgery, was found to be feasible and safe, with high adherence and no major adverse effects. Additionally, hospital stay and the time from surgery to chemotherapy were lower in the prehabilitation cohort than in the cohort of historical controls, considering that both groups had received perioperative care in accordance with ERAS guidelines. 

### 4.2. Results in the Context of Published Literature

Patients with AOC who are candidates for cytoreductive surgery often present considerable cognitive and physical deterioration in the context of malnourishment [14]. Consequently, a high-intensity programme of interval training within a short time frame before surgery in these fragile patients might be considered unfeasible. Contrarily, we found that this population presented high adherence to the supervised exercise sessions, with no major adverse effects preventing the continuation of training exercises. This might be explained in that supervised structured exercises encourage patient adherence despite poor baseline functional status. Although some studies in patients undergoing other abdominal surgeries used easier alternatives, such as walking-based interventions performed at home, these approaches depend on self-reporting data and therefore, adherence rates may be unreliable [12,23].

Whether short, high-intensity exercise programmes can produce sufficient improvement is controversial. In previously reported studies, the duration of prehabilitation in non-gynaecological major abdominal surgeries ranged from 2 to 6 weeks [13]. Nevertheless, in patients with AOC who are candidates for PCS, it is important to complete the prehabilitation programme within the shortest possible period of time to avoid tumour progression and detrimental effects on oncologic outcomes [7,24]. However, most patients with AOC usually undergo neoadjuvant chemotherapy, making it possible to plan and carry out an appropriate programme within an optimal timeframe. In a systematic review analysing the effects of a preintervention exercise programme in patients undergoing abdominal surgery, Moran et al. [12] concluded that prehabilitation in patients treated with neoadjuvant chemotherapy is safe and can increase aerobic capacity before surgery. In line with these results, in our study, we observed high adherence to the exercise programme in the patients who received neoadjuvant treatment. 

Therefore, despite the baseline status of these patients and regardless of whether they are receiving neoadjuvant chemotherapy, we showed that undergoing a prehabilitation programme seems feasible in this population.

Adherence to the second component of our multimodal prehabilitation programme involving the ingestion of preoperative nutritional complements and immunomodulatory preparations was excellent. Actually, we found that patients in the prehabilitation cohort had significantly better prealbumin levels than the control cohort. A prospective randomised study in non-malnourished patients undergoing surgery for abdominal cancer found that nutritional supplementation for 14 days before surgery significantly reduced the number and severity of postoperative complications [25]. Similarly, another study showed that the administration of immune-enhancing proteins reduced complication rates [26]. 

Adherence to the third component of our multimodal prehabilitation programme, psychological support, was also high. No firm evidence is available on the effectiveness of employing anxiety-reduction strategies before surgery. A Cochrane review found only low-quality evidence supporting psychological preparation for postoperative pain, behavioural recovery, and length of hospital stay [27]. Thus, we can only speculate that these methods helped patients in our programme to deal with the stressful preoperative period and reinforced adherence to the physical and nutritional interventions.

There is no consensus in the literature on how to measure the potential benefits of prehabilitation programmes [9,28,29]. In a blinded randomised controlled trial performed at the Hospital Clinic of Barcelona to assess the impact of a prehabilitation intervention on perioperative complications in high-risk patients undergoing major abdominal surgery, Barberan-Garcia et al. [30] found a lower rate of postoperative complications (31% in the prehabilitation group vs. 62% in controls; *p* = 0.001), demonstrating the protective role of the intervention against postoperative complications. To analyse postoperative complications, we used the CCI to report the overall morbidity of patients after surgery. The Clavien–Dindo classification [2], which describes five grades of severity for the most known complications, uses only the single most severe complication occurring in a patient during a given episode of care. Instead, the CCI reports all postoperative complications in a tabulated form and seems to be more sensitive than existing morbidity endpoints [22]. To our knowledge, this is the first study to use this more robust approach in an AOC population. The rate of complications in the control cohort was similar to that previously described in the literature [3] and higher, albeit not significantly different, compared to the prehabilitation cohort. Nevertheless, this was not the primary objective of the study, and given the limited number of patients, our population did not have enough events to allow the detection of a difference if one actually existed. 

In our study, the hospital length of stay was significantly lower in the prehabilitation cohort, irrespective of the fact that both groups received perioperative care in accordance with ERAS guidelines. Hospital length of stay seems to be an appropriate indicator of the effect of prehabilitation, because it reflects the extent to which patients have recovered baseline physical functioning; moreover, shorter stays likely result in lower hospital costs [31]. Nevertheless, other studies have reported discrepant results regarding the effects of prehabilitation on hospital stay [13,32,33]. These discrepancies might be explained by multiple factors, such as the heterogeneity of operative procedures and postoperative care protocols. Most reports fail to mention whether ERAS care pathways were used for postoperative care. Finally, non-medical reasons could influence when patients are discharged from the hospital. Future studies should use standardised postoperative care pathways to enable comparison among studies.

We also analysed the effect of prehabilitation on the length of time before starting chemotherapy. We found that the time interval from surgery to starting chemotherapy was significantly lower in the prehabilitation cohort than in the control cohort. The optimal time between debulking surgery and the start of subsequent chemotherapy has not been established, but it is recommended to be as short as possible and no longer than 4–6 weeks [34]. Shorter time to starting chemotherapy could directly improve the prognosis of the patient, since delays in initiating systemic therapy are associated with decreased survival in patients with AOC [7]. 

### 4.3. Strengths and Weaknesses

One major strength of our study is our centre’s specialised coordinated multidisciplinary group, which has accumulated extensive experience in prehabilitation and has achieved encouraging results in non-gynaecological surgery. Moreover, patients diagnosed with AOC stand to benefit more from prehabilitation than patients with other gynaecological cancers since they are at a high risk of postoperative complications and functional decline after surgery. We designed a multimodal prehabilitation programme to maximise the impact on functional outcomes, as multimodal programmes are more effective than single-mode prehabilitation interventions [11]. Finally, to minimise differences in perioperative care and to make our results easier to compare with future studies, we used ERAS^®^ pathways in both cohorts.

We do recognise some limitations in our study. This was a pilot study including a low number of patients at a single institution, and thus, caution is warranted in extrapolating our results to other contexts. The non-randomised study design is a major limitation, since the historical control cohort might not have received the same attention from the staff as the prospective prehabilitation cohort, and thus, this could have benefitted the latter population. We also admit this might represent a heterogeneous group of patients since they are treated at various points regarding their cancer journeys. Lastly, although the same preoperative, surgical, and postoperative protocols were followed in both cohorts, they were not matched.

### 4.4. Implications for Practice and Future Research

This preliminary study shows that multimodal prehabilitation is feasible, safe, and effective in patients with AOC. These results are significant since they have not been previously reported in patients with AOC who undergo cytoreductive surgeries; nonetheless, as this is a pilot study, caution is recommended. This approach must be further evaluated in larger, randomised trials involving multiple centres. Along these lines, our results have led us to design a multicentre, randomised controlled trial, called SOPHIE (Surgery in Ovarian cancer with Pre-Habilitation In ERAS environment), which is registered with ClinicalTrials.gov (number NCT 04862325) and began recruiting patients in 2021.

## 5. Conclusions

A multimodal prehabilitation programme before cytoreductive surgery is feasible in AOC patients, resulting in no major adverse effects and a significantly shorter hospital stay and time to starting chemotherapy.

## Figures and Tables

**Table 1:** Clinical and surgical characteristics of patients who underwent surgery for advanced ovarian cancer: multimodal prehabilitation vs. matched controls.

Characteristics	Prehabilitation Cohort	Control Cohort	*p*-Value
	*n* = 15	*n* = 19	
Age (years), median [IQR]	55 (52–69)	60 (52–72)	0.5
Body mass index (kg/m^2^), median [IQR]	25 (23–26)	24 (24–26)	0.36
Charlson Comorbidity Index, median [IQR]	4 (3–5)	4 (3–6)	0.8
ECOG, *n* (%)			
0	10	6	0.08
1	5	13	
CA 125, median [IQR]	30 (16–166)	76 (30–585)	0.09
Preoperative albumin (g/L), median [IQR]	44 (43–46)	44 (36–46)	0.3
FIGO staging, *N* (%)			0.029
III B	1 (6.7)	0 (0)	
III C	5 (33.4)	12 (63.2)	
IV A	4 (26.7)	0 (0)	
IVB	5 (33.4)	7 (36.8)	
Histology, *n* (%)			
Low-grade serous carcinoma	1 (6.7)	2 (10.5)	
High-grade serous carcinoma	12 (80)	16 (84.2)	
Endometrioid carcinoma	1 (6.7)	1 (5.3)	0.87
Granulosa cell tumour	1(6.7)	0 (0)	
Duration of surgery (min), median [IQR]	320 (255–280)	310 (270–260)	0.4
PCI, median [IQR]	10 (8–14)	10 (8–14)	0.97
Type of surgery, *n* (%)			
Primary cytoreduction surgery	1 (6.7)	7 (36.8)	
Interval debulking surgery	11 (73.3)	11 (57.9)	0.21
Secondary or tertiary cytoreduction	3 (0.2)	1 (5.2)	
Procedure, *n* (%)			
Hysterectomy + bilateral salpingo-oophorectomy	11 (73.3)	18 (94.73)	0.14
Pelvic peritonectomy	6 (40)	5 (26.31)	0.475
Intestinal resection	7 (46.66)	10 (52.63)	1
Colorectal resection	5	3	1
Large bowel resection	3	5	1
Small bowel resection	3	5	0.69
Appendectomy	4 (26.67)	6 (31.58)	1
Radical omentectomy	12 (80)	17 (89.47)	0.63
Partial hepatectomy	1 (6.67)	0 (0)	0.44
Splenectomy	2 (13.3)	1 (5.26)	0.57
Diaphragmatic stripping	7 (46.67)	4 (21.05)	0.15
HIPEC	1 (7.14)	1 (5.26)	1
Debulking of pelvic/paraaortic lymph nodes	10 (66.67)	14 (77.78)	0.69
Aletti Complexity Score, median [IQR]	6 (4–8)	7 (4–8)	0.51
Residual disease, *n* (%)			
R0	13 (86)	19 (100)	
R1	1 (6.7)	0 (0)	0.18
R2	1 (6.7)	0 (0)	

IQR: interquartile range; ECOG: Eastern Cooperative Oncology Group; PCI: peritoneal cancer index; HIPEC: hyperthermic intraperitoneal chemotherapy; CA: cancer antigen.

**Table 2:** Prehabilitation programme adherence and adverse effects.

Characteristics	Prehabilitation Cohort
Duration of programme (weeks), median weeks [IQR]	2 (2,3)
Number of gym sessions, median [IQR]	6 (4–8)
Adherence to gym sessions: Patients completing >2 sessions or >75% of scheduled sessions, *n* (%)	13 (86.6)
Adherence (%), median [IQR]	83 (60–100)
Adverse effects, *n* (%)	3/15 (20)
Mild dizziness	3/15 (20)
Adherence to nutrition intervention >75%, *n* (%)	15 (100)
Adherence to psychological intervention ≥1 session, *n* (%)	12 (80)

IQR: interquartile range.

**Table 3:** Surgical and oncological outcomes of patients with advanced ovarian cancer treated with or without multimodal prehabilitation.

Characteristics	Prehabilitation Cohort	Control Cohort	*p*-Value
	*n* = 15	*n* = 19	
Hospital stay (days), median [IQR]	5 (4–6)	7 (5–9)	0.041
Intensive care unit stay, days (%)			
0	9 (60)	9 (47.4)	
1	5 (33.3)	6 (31.6)	
2	1 (6.7)	3 (15.8)	0.88
3	0 (0)	1 (5.3)	
Preoperative prealbumin (g/L), median [IQR]	0.235 (0.214–0.316)	0.180 (0.13–0.23)	0.007
Intraoperative complications, *n* (%)			
Intestinal injury	0 (0)	1 (5.3)	
Vascular injury	0 (0)	0 (0)	
Urological injury	0 (0)	0 (0)	0.40
Nerve injury	0 (0)	0 (0)	
Need for blood transfusion	1 (6.7)	5 (26.3)	
CCI, median [IQR]	0 (0–20.9)	8, 66 (0–33.5)	0.20
CCI Mean, SD	9, 33 (12.1)	16, 62 (16.9)	0.08
Clavien–Dindo classification <30 days, median [IQR]			
Patients with ≥1 complication	6 (40)	12 (63.15)	
I	4 (40)	4 (23.5)	0.30
II	6 (60)	8 (47)	
III a	0 (0)	2 (11.8)	
III b	0 (0)	2 (11.8)	
IV a	0 (0)	1 (5.9)	
IV b	0 (0)	0 (0)	
V	0 (0)	0 (0)	
Major complications (Clavien–Dindo ≥ III), *n* (%)			
No	15 (100)	14 (73.7)	0.053
Yes	0 (0)	5 (26.3)	
Type of complications, *n* (%)			
Paralytic ileus/intestinal obstruction	4 (40)	1 (5.3)	
Cardiovascular complications	0 (0)	1(5.3)	
Pulmonary complications/DVT/PE	0 (0)	0 (0)	
Anastomotic leakage/peritonitis	0 (0)	0 (0)	
Infection/postsurgical abscess	3 (30)	5 (26.3)	0.714
Postsurgical bleeding/need for transfusion	2 (20)	9 (47.3)	
Lymphocele/lymphatic complications	0 (0)	3 (15.8)	
Time to starting chemotherapy (days), median [IQR]	25 (23–35)	35 (28–45)	0.03

DVT: deep vein thrombosis; PE: pulmonary embolism (PE); CCI: comprehensive complication index; IQR: interquartile range.

## Data Availability

Data sharing is not applicable to this article.
